# Granulomatosis with Polyangiitis (GPA) in a Polish Tertiary Centre (2010–2025): Sex-Stratified Phenotypes, Serology, and Evolving Treatment Patterns

**DOI:** 10.3390/jcm14217884

**Published:** 2025-11-06

**Authors:** Aleksandra Hus, Małgorzata Wisłowska, Krzysztof Bonek

**Affiliations:** Rheumatology Clinic of the National Institute of Geriatric, Rheumatology and Rehabilitation, 02-637 Warsaw, Poland; aleksandra.hus@spartanska.pl (A.H.); krzysztof.bonek@spartanska.pl (K.B.)

**Keywords:** granulomatosis with polyangiitis, ANCA-associated vasculitis, PR3-ANCA, subglottic stenosis, diffuse alveolar haemorrhage, rituximab, avacopan

## Abstract

**Background/Objectives**: GPA is a PR3-ANCA–predominant small vessel vasculitis with organ involvement. Real-world, single-centre data are needed to interpret evolving therapies and phenotype patterns in national conditions. **Material and Methods**: Retrospective cohort study of consecutive GPA patients managed at the National Institute of Geriatrics, Rheumatology and Rehabilitation (Warsaw, Poland) from 1 September 2010 to 1 September 2025. Data included demographics, phenotype, BVAS, organ involvement, PR3/MPO-ANCA serology, and induction/maintenance therapies. **Results**: Fifty patients were included (54.0% men). Mean age was 52.5 years; mean BMI was 26.15 kg/m^2^. Ear-nose-throat (ENT) disease was frequent: rhinosinusitis 76.0%, nasal cartilage destruction 64.0%, subglottic stenosis 34.0%. Pulmonary nodules occurred in 52.0%, cavitation in 28.0%, and diffuse alveolar haemorrhage in 34.0%. Renal involvement included haematuria in 42.0%, chronic kidney disease (CKD) in 32.0%, and rapidly progressive kidney disease in 22.0%. Orbital inflammation was 36.0%, and PR3-ANCA was positive in 70.0%. All patients received glucocorticoids for induction; cyclophosphamide 28/50 (56.0%), rituximab 6/50 (12.0%), and mycophenolate with methotrexate 6/50 (32%). Maintenance therapy included methotrexate (78.0%), mycophenolate (64.0%), rituximab (52.0%), and azathioprine (12.0%). **Conclusions**: This Polish single-centre cohort shows an ear-nose-throat-lung-kidney (ELK)-dominant, PR3-predominant GPA phenotype and frequent but variable kidney involvement. Over 2010–2025, practice changed toward rituximab-based strategies, steroid minimisation, selective use of plasma exchange, and early avacopan uptake, with tofacitinib for maintenance therapy as a possible new therapeutic option.

## 1. Introduction

Granulomatosis with polyangiitis (GPA) is a necrotising granulomatous inflammation affecting small-to-medium-sized vessels, classified within the spectrum of ANCA-associated vasculitides (AAV). It is most frequently associated with anti-proteinase 3 (PR3-ANCA) seropositivity and is defined according to the weighted 2022 ACR/EULAR classification criteria [1].

The previous 1990 GPA classification criteria [2] have now been replaced by the 2022 criteria. The 1990 ACR criteria included only nasal or oral inflammation, abnormal chest radiograph, urinary sediment, and granulomatous inflammation on biopsy [2]. The 2022 ACR/EULAR criteria for GPA significantly improved the diagnosis because they combined cartilaginous involvement of ear or nose cartilage, hoarse voice or stridor, endobronchial involvement, saddle nose deformity, and conductive or sensorineural hearing loss. They differ in their specific scoring and how they weight factors such as imaging, biopsy results and positive antibody tests.

There are two differences between the 2022 ACR/EULAR criteria and the 1990 ACR criteria. One is that the new criteria include items classified into clinical, laboratory, radiological and histological categories [3]. The second is that they assign differently weighted points to each item, and they provide cut-off values of the total score for the classification of GPA [3].

AAV demonstrates notable epidemiological heterogeneity. Incidence is higher in Northern and Western Europe (approximately 8–12 per million per year) compared to Southern Europe and parts of Asia, reflecting a gradient wherein PR3-ANCA is predominant in northern latitudes and MPO-ANCA is more common in Eastern Asia and Southern Europe [4,5]. Polish registry data (POLVAS) confirm this national cohort aligns with Central/Northern European patterns, characterised by PR3-ANCA predominance and a classic ELK (ear, nose, throat, lung, and kidney) phenotype with frequent ENT and pulmonary involvement [6]. This geographical variation, alongside genetic risk alleles and environmental triggers such as Staphylococcus aureus carriage, underscores the value of national cohort studies for interpreting trial evidence and understanding phenotypic heterogeneity [7,8]. These epidemiological and clinical benchmarks provide a framework for interpreting organ-specific frequencies in single-centre cohorts.

### Aim

This study aimed to characterise the demographic, clinical, serological, and therapeutic profiles of GPA patients managed at a national tertiary referral centre in Poland over a 15-year period, with a specific analysis of organ manifestation and treatment patterns stratified by sex.

## 2. Methods and Materials

A single-centre, retrospective cohort study was conducted at the Rheumatology Clinic of the National Institute of Geriatrics, Rheumatology and Rehabilitation in Warsaw, Poland. The study included consecutive patients with GPA managed between 1 September 2010 and 1 September 2025. Diagnosis was confirmed according to the 2022 ACR/EULAR classification criteria (score ≥ 5) based on clinical, serological, imaging, and histopathological data [1].

### 2.1. Data Collection

Data abstraction from medical records included:

- Demographics: age, sex, and body mass index (BMI).
- Disease characteristics: time of observation, phenotype (generalised, localised, and renal-predominant), and disease activity assessed by the Birmingham Vasculitis Activity Score (BVAS).
- Organ system involvement: comprehensive data were collected on ENT/pulmonary, renal, ocular, cutaneous, neurological, and cardiac manifestations.
- Serology: PR3-ANCA and MPO-ANCA status.
- Treatment: induction therapies (glucocorticoids, cyclophosphamide, rituximab, and mycophenolate with methotrexate) and maintenance therapies (rituximab, azathioprine, mycophenolate mofetil, methotrexate, tofacitinib, and avacopan).

### 2.2. Data Analysis

Categorical variables were coded binarily (1 = present, 0 = absent; sex: 3 = male, and 2 = female). Descriptive statistics were employed: continuous variables are presented as median and range, and categorical variables as counts and percentages (*n*, %). The cohort consisted of 50 patients (27 men and 23 women), and all analyses are descriptive due to the sample size.

### 2.3. Ethical Considerations

The study protocol adhered to the principles of the Declaration of Helsinki and Good Clinical Practice. Approval was granted by the institutional bioethics committee (KBT-6/10/2022, 16 November 2022). Written informed consent was obtained from all participants.

## 3. Results

The cohort comprised 50 patients (54.0% men and 46.0% women). The mean age among males was 51.4 years (median 52; 20–77) and 53.7 years, (median 51; range 20–85) among females. The mean BMI was 26.89 kg/m^2^ in men and 25.29 kg/m^2^ in women. The other clinical data, such as the number of individuals with specific initial disease manifestations due to EUVAS classification and median BVAS score at the diagnosis, are presented in Table 1. The detailed system manifestations were divided into two forms—first into presentation in both sex groups, second due to serological status (ANCA+/−) of patients; it can be found in Table 2 and Table 3. While their detailed description with the female/male and serology participation can be found in the text.

### 3.1. ENT and Airway

There was at least one otorhinolaryngological and upper airway involvement in every patient. Disease manifestations were overlapping and were observed as rhinosinusitis, nasal/oral ulceration, destructive nasal cartilage lesions leading to nasal deformity, subglottic/tracheal stenosis, conductive/auditory involvement, and recurrent otitis media. Rhinosinusitis alone was documented in 38 patients (19 male and 19 female), indicating that upper airway disease affected both sexes. The detailed distribution of the ENT and airway manifestation of GPA can be found in Table 2.

### 3.2. Pulmonary

Pulmonary involvement was also frequent in this cohort. Clinical and radiologic evidence of lung disease (with different overlap), including pulmonary nodules, cavitation, diffuse/alveolar haemorrhage, bronchiectasis, and interstitial lung disease, was identified in at least 26 patients (52% of the cohort), showing that pulmonary disease was a common manifestation in our series. Pulmonary nodules were the most common abnormality and were documented in 26 patients (18 male and 8 female), and these were often accompanied by cavitation (14 patients; 10 male and 4 female). Diffuse/alveolar haemorrhage occurred in 17 patients (11 male and 6 female), while bronchiectasis was noted in 11 patients (5 male and 6 female). Interstitial lung disease was uncommon, identified in three patients and exclusively in men. Details are shown in Table 2.

### 3.3. Renal Involvement

Overall, renal involvement affected 68% of patients, depending on how overlapping phenotypes are defined. Chronic kidney disease affected 42% of the cohort (M = 57% and F = 43%), and microscopic haematuria was present in 36% (M = 56% and F = 44%). Rapidly progressive glomerulonephritis and nephritic syndrome were each observed in 22% of patients and showed a male predominance (M = 64% and F = 36%). This reflects a spectrum of kidney injury ranging from isolated haematuria to chronic kidney impairment and rapidly progressive glomerulonephritis.

### 3.4. Different Organs: Ocular/Orbital Disease/Cutaneous/Musculoskeletal Involvement

Orbital inflammatory disease was observed in 18 patients (36% of the cohort; 10 males and 8 females). Cutaneous involvement affected between 13 and 25 patients (26–50% of the cohort), including purpura, skin ulceration, and livedo reticularis. Musculoskeletal involvement (arthralgia and/or myalgia) was very common, affecting at least 27 patients (54%) and possibly up to most of the cohort.

### 3.5. Cardiovascular Involvement

Cardiovascular events were also systematically recorded in this cohort. Cardiac involvement was less frequent, with pericarditis documented in seven patients (14%). In contrast, vascular complications—including venous thromboembolism and cerebrovascular events—were more common, affecting between 11 and 21 patients (22–42% of the cohort).

### 3.6. Serological Status

Serological findings (ANCA) are summarised in Table 3. PR3-ANCA positivity was predominant (70.0%), while MPO-ANCA was rare (2.0%). ANCA was negative in 28.0% of patients.

The frequency of organ involvement and specific clinical manifestations between ANCA-positive and ANCA-negative GPA patients can be found in Table 3. Overall, ENT involvement is clearly more prominent in the ANCA-positive group. Rhinosinusitis, nasal/oral ulceration, destruction of nasal cartilage with resulting nasal deformity, and subglottic or tracheal stenosis all occur substantially more often in ANCA-positive patients. Airway obstruction shows the same pattern. Hearing loss and recurrent otitis media are present in both groups at comparable levels. Pulmonary manifestations show a mixed picture. Alveolar haemorrhage and cavitating lung lesions are mainly seen in ANCA-positive patients, whereas interstitial lung disease appears relatively more often in ANCA-negative patients. Pulmonary nodules and bronchiectasis occur in both groups. Renal involvement is frequent in both ANCA-positive and ANCA-negative patients. Rapidly progressive renal disease and nephritic syndrome are more typical of ANCA-positive cases, but haematuria and chronic kidney disease are common in both groups, and haematuria is slightly more frequent in ANCA-negative patients. Orbital inflammatory disease is seen in both groups, with a modest predominance in ANCA-positive patients. Cutaneous manifestations such as purpura and skin ulcers are mostly confined to ANCA-positive patients; they are rare or absent in ANCA-negative patients. Vascular events (venous thromboembolism and cerebrovascular events) occur in both groups. Venous thromboembolism appears somewhat more frequent in ANCA-negative patients. Musculoskeletal symptoms (arthralgias and myalgia) are markedly more common in ANCA-positive patients. Cardiac involvement in the form of pericarditis is relatively uncommon and occurs at a similar low frequency in both groups.

### 3.7. Histopathology Results

Histopathological findings are summarised in Table 4. Renal biopsies demonstrated glomerulonephritis. Nasal and paranasal sinus biopsies were predominantly characterised by necrotising inflammation and vasculitis. Lung biopsies most often showed granulomas and vasculitis as the main pathological processes. Skin biopsies consistently revealed necrotic and vasculitic changes. Histopathological material was available for patients who underwent renal biopsy, nasal or paranasal sinus biopsy, lung biopsy, and skin biopsy.

### 3.8. Treatment

Induction therapy included cyclophosphamide, rituximab, and mycophenolate mofetil. All patients received glucocorticoids during induction therapy. Plasma exchange was used only once. For maintenance therapy, methotrexate was the most frequently used agent, followed by mycophenolate mofetil, rituximab, and azathioprine.

A subset of patients received newer therapies, including avacopan and tofacitinib, which were used as options in non-responsive cases. The safety profile and disease control were acceptable. Maintenance therapy was often modified during follow-up, and most patients were treated with combinations of two immunosuppressive drugs. Treatment characteristics are summarised in Table 5.

## 4. Discussion

This single-centre cohort from a Polish tertiary clinic demonstrates a GPA phenotype characterised by a high ENT and airway burden, substantial pulmonary involvement, kidney disease, and a predominance of PR3-ANCA. Clinically, ENT manifestations are often the initial and most prevalent feature and tend to lead not only to local complications such as septal perforation and saddle-nose deformity but also to severe, potentially life-threatening processes, including temporal bone infiltration [9]. Subglottic stenosis (SGS) is a critical airway complication, affecting 10–23% of patients, with a recognised predilection for women and younger individuals [10,11]. Pulmonary involvement, typically presenting as multiple nodules on computed tomography, often with cavitation, is another hallmark pattern being found in 66–85% of cases; in contrast, diffuse alveolar haemorrhage (DAH) is less common but represents a life-threatening presentation [9,12]. Kidney disease remains a major prognostic determinant, developing in 70–85% of patients, frequently manifesting as pauci-immune necrotising and crescentic glomerulonephritis [13]. Ocular involvement ranges from limited anterior inflammation to vision-threatening or orbital disease [14].

First in our study, airway and ENT disease was prominent, and subglottic stenosis displayed a clear female preponderance. Observations from this study are consistent with otolaryngology-focused series describing clinically important airway lesions [11]. Locoregionally aggressive disease presents a high risk of relapse despite systemic treatment. These observations align with the recognition that granulomatous airway disease can follow a course only partly concordant with systemic activity. Those observations reinforce the need for proactive airway surveillance and procedural access alongside aggressive treatment. On the contrary, lung involvement such as nodules, lung cavitations and alveolar haemorrhage were more common in male patients. Widely described in literature, interstitial lung disease occurred only in three male patients with long disease progression [15].

Second, while renal involvement was common, we observed a spectrum from microscopic haematuria to rapidly progressive glomerulonephritis rather than severe kidney failure at presentation. Chronic kidney disease was observed frequently; however, its origin was not only related to the vasculitis but could also develop because of the age of patients, comorbidities and pharmacotherapy used by the patients [16].

Regarding ANCA serology, most patients in this cohort were PR3-ANCA positive. ANCA-positive patients more often demonstrated classic GPA features: destructive ENT disease (including cartilage loss and nasal deformity), subglottic or tracheal stenosis, and airway obstruction. They also more frequently had pulmonary nodules, cavitation, and alveolar haemorrhage. In contrast, ANCA-negative patients displayed proportionally more interstitial lung disease and showed a similar or even higher rate of haematuria despite the absence of circulating ANCA. These patterns indicate that serotype (ANCA-positive vs. ANCA-negative) and sex both indicate distinct organ-dominant phenotypes. Clinically, this argues for ANCA-aware and sex-aware monitoring strategies: for example, structured airway follow-up (including scheduled laryngotracheal evaluation) in women with ENT-dominant disease and dedicated thoracic imaging surveillance in men with nodular/cavitating lung involvement.

Therapeutically, rituximab has become the best agent of both induction and maintenance therapy in our practice, according to EULAR recommendations [17]. This approach enables glucocorticoid tapering and, in many cases, avoidance or minimisation of high-dose cyclophosphamide exposure in induction. The C5a receptor inhibitor avacopan has recently entered clinical use as a steroid-sparing adjunct in severe disease, supported by data from the ADVOCATE trial [18]. In selected highly active or refractory cases, we also introduced tofacitinib in combination with rituximab during maintenance therapy. In most of these patients, disease control and safety profiles were acceptable, although one patient developed a thrombotic complication and another did not respond to tofacitinib after several months, leading to treatment discontinuation. These experiences underline that escalation beyond standard regimens is sometimes feasible and clinically meaningful but must be individualised and closely monitored. Our study has limitations common to single-centre retrospective analyses: referral bias towards complex cases, incomplete histology in localised disease, and limited power for adjusted sex-specific or ANCA-specific interaction analyses.

There are three practical directions which emerge from these data. First, surveillance pathways should be tailored to disease pattern: early ENT/airway involvement in women may justify routine endoscopic airway assessment and low-threshold procedural intervention; conversely, men with nodular/cavitating lung disease may benefit from structured thoracic CT follow-up to detect progression or haemorrhage. Second, treatment algorithms in GPA are clearly moving toward glucocorticoid-sparing strategies, preferred rituximab, often in combination with a second immunosuppressive agent such as tofacitinib, rather than monotherapy. Third, the role of newer agents such as avacopan—and, in resistant cases, JAK inhibition—should be prospectively evaluated in relation to serotype (PR3-/MPO-/ANCA-negative), sex, and organ-dominant phenotype, including airway vs. lung parenchyma vs. kidney.

## 5. Conclusions

This study of 50 GPA patients confirms a classic PR3-ANCA dominant phenotype with characteristic ELK organ involvement. The cohort showed predominant ENT and pulmonary manifestations, with glomerulonephritis. ANCA-positive patients exhibited more severe organ involvement, particularly destructive ENT lesions and pulmonary complications. Treatment patterns reflected contemporary approaches with high rituximab therapy and emerging complement inhibition. These findings emphasise the need for multidisciplinary, serotype-aware management in GPA care.

## Figures and Tables

**Table 1:** Clinical and demographic characteristics of patients with GPA.

Characteristic	Overall (*N* = 50)	Men (*n* = 27)	Women (*n* = 23)
**Age, years**			
Mean	52.4	51.4	53.7
Median	51	52	45
Range	20–85	20–77	20–85
**BMI**			
Mean	26.2	26.9	25.3
Median	25.35	26.4	24.0
Range	19.0–30.5	24.0–30.5	19.0–29.5
**Initial classification according to EUVAS**			
Localised	12 (24.0%)	4 (14.8%)	8 (34.8%)
Systemic	21 (42.0%)	8 (29.6%)	13 (56.5%)
Generalised	13 (26.0%)	8 (29.6%)	4 (17.4%)
Severe renal	4 (8.0%)	3 (11.1%)	2 (8.7%)
**BVAS at diagnosis**			
Median	13	13	13

**Table 2:** Organ system involvement in GPA patients (*N* = 50).

System/Domain	Manifestation	Total *n* (%)	Male *n* (%)	Female *n* (%)
**ENT/Upper airway**	**Any ENT/upper airway involvement**	**38–50 (76.0–100.0)**	**-**	**-**
	Rhinosinusitis	38 (76.0)	19 (38.0)	19 (38.0)
	Nasal or mouth ulcers	34 (68.0)	18 (36.0)	16 (32.0)
	Destruction of nasal cartilage	32 (64.0)	15 (30.0)	17(34.0)
	Nasal deformity	24 (48.0)	10 (20.0)	14 (28.0)
	Subglottic or tracheal stenosis	17 (34.0)	6 (12.0)	11 (22.0)
	Hearing loss	22 (44.0)	10 (20.0)	12 (24.0)
	Airway obstruction	13 (26.0)	8 (16.0)	5 (10.0)
	Nasal polyps	3 (6.0)	1 (2.0)	2 (4.0)
	Recurrent otitis media	15 (30.0)	8 (16.0)	7 (14.0)
**Pulmonary**	**Any pulmonary involvement**	**26–50 (52.0–100.0)**	**-**	**-**
	Pulmonary nodules	26 (52.0)	18 (36.0)	8 (16.0)
	Cavitation	14 (28.0)	10 (20.0)	4 (8.0)
	Diffuse/alveolar hemorrhage	17 (34.0)	11 (22.0)	6 (12.0)
	Bronchiectasis	11 (22.0)	5 (10.0)	6 (12.0)
	Interstitial lung disease	3 (6.0)	3 (6.0)	0 (0.0)
**Renal**	**Any renal involvement**	**21–34 (42.0–68.0)**	**-**	**-**
	Chronic kidney disease	21 (42.0)	12 (24.0)	9 (18.0)
	Microscopic hematuria	18 (36.0)	10 (20.0)	8 (16.0)
	Rapidly progressive glomerulonephritis (RPGN)	11 (22.0)	7 (14.0)	4 (8.0)
	Nephritic syndrome	11 (22.0)	7 (14.0)	4 (8.0)
**Orbital/Ocular**	Orbital inflammatory disease	18 (36.0)	10 (20.0)	8 (16.0)
**Cutaneous**	**Any cutaneous involvement**	**13–25 (26.0–50.0)**	**-**	**-**
	Purpura	13 (26.0)	7 (14.0)	6 (12.0)
	Skin ulcers	8 (16.0)	5 (10.0)	3 (6.0)
	Livedo reticularis	4 (8.0)	3 (6.0)	1 (2.0)
**Vascular**	**Any vascular involvement**	**11–21 (22.0–42.0)**	**-**	**-**
	Venous thromboembolism	10 (20.0)	6 (12.0)	4 (8.0)
	Cerebrovascular event	11 (22.0)	7 (14.0)	4 (8.0)
**Cardiac**	**Any cardiac involvement**	**7 (14.0)**	**-**	**-**
	Pericarditis	7 (14.0)	6 (12.0)	1 (2.0)

**Note:** Percentages are calculated with respect to the total cohort (*N* = 50). “Any [system] involvement” refers to the presence of ≥1 manifestation listed within that system. Manifestations within a system are not mutually exclusive and may coexist in the same patient; therefore, row counts within a system do not sum to the “Any [system] involvement” range and can collectively exceed the total cohort size. Ranges indicate the minimum and theoretical maximum number of unique affected patients, depending on overlap between manifestations. Syndromes included in one group can overlap.

**Table 3:** Comparison of clinical manifestations between ANCA positive and ANCA negative GPA patients.

Variable	ANCA+ (*n* = 36, 72%)		ANCA− (*n* = 14, 28%)	
**ANCA serotype distribution**				
PR3-ANCA	35/36	97.2%	-	-
MPO-ANCA	1/36	2.8%	-	-
**ENT involvement (*n* = 50)**				
Rhinosinusitis	32/36	88.9	6/14	42.9
Destruction of nasal cartilage	26/36	72.2	6/14	42.9
Nasal deformity	19/36	52.8	5/14	35.7
Subglottic or tracheal stenosis	16/36	44.4	1/14	7.1
Hearing loss	16/36	44.4	6/14	42.9
Airway obstruction	12/36	33.3	1/14	7.1
Nasal polyps	3/36	8.3	0/14	0
Recurrent otitis media	9/36	25.0	6/14	42.9
**Lung involvement (*n* = 50)**				
Alveolar hemorrhage	15/36	41.7	2/14	14.3
Pulmonary nodules	20/36	55.5	6/14	42.8
Interstitial lung disease	1/36	2.8	2/14	14.3
Bronchiectasis	8/36	22.2	3/14	21.4
Cavitation	14/36	38.9	0/14	0
**Renal involvement (*n* = 34)**				
Rapidly progressive renal disease	10/36	27.7	1/14	7.1
Nephritic syndrome	10/36	27.7	1/14	7.1
Chronic kidney disease	12/36	33.3	4/14	28.6
Hematuria	14/36	38.9	7/14	50.0
**Orbital involvement (*n* = 18)**				
Orbital Inflammatory Disease	14/36	38.9	4/14	28.6
**Cutaneous involvement (*n* = 25)**				
Purpura	12/36	33.3	1/14	7.1
Skin ulcers	8/36	22.2	0/14	0
Livedo reticularis	4/36	11.1	0/14	0
**Vascular involvement (*n* = 21)**				
Venous thromboembolism	6/36	16.7	4/14	28.6
Cerebrovascular event	8/36	22.2	3/14	21.4
**Musculoskeletal involvement**				
Arthralgias	24/36	66.7	3/14	21.4
Myalgia	21/36	58.3	3/14	21.4
**Cardiac involvement (*n* = 7)**				
Pericarditis	5/36	13.9	2/14	14.2

**Legend:** *n*—number of patients with specific feature within group; *N*—total number of ANCA-positive (*n* = 36) or ANCA-negative (*n* = 14) patients; %—percentage of patients with specific feature within each ANCA group. Numbers in parentheses after system names indicate total number of patients with involvement of that organ system (based on overall cohort data). **Study population:** Total cohort *N* = 50 patients. ANCA-positive: 36 patients (72%), including PR3-ANCA: 35 patients (97.2% of ANCA+ group, 70% of total cohort) and MPO-ANCA: 1 patient (2.8% of ANCA+ group, 2% of total cohort). ANCA-negative: 14 patients (28% of total cohort).

**Table 4:** Histopathological characteristics of patients with GPA.

Histopathological Finding	*n*	%
**Renal biopsy (Glomerulonephritis *n* = 15)**		
Crescentic glomerulonephritis	6	10%
Pauci-immune crescentic glomerulonephritis	5	10%
Focal segmental glomerulonephritis	4	8%
**Nasal/paranasal sinuses biopsy (*n* = 35)**		
Necrotizing inflammation	35	70%
Granulomas	20	40%
Vasculitis	35	70%
**Lung biopsy (*n* = 8)**		
Granulomas	6	12%
Vasculitis	6	12%
**Skin biopsy (*n* = 2)**		
Necrosis	2	4%
Vasculitis	2	4%

**Note:** Percentages are calculated with respect to the total cohort (*N* = 50 patients).

**Table 5:** Treatment characteristics of patients with GPA (*N* = 50).

Treatment	*n* (%)	Women (*n* = 23)	Men (*n* = 27)
**Induction therapy**			
Glucocorticoids	50 (100%)	100%	100%
Cyclophosphamide	28 (56.0%)	52.2%	59.3%
Methotrexate and Mycophenolate mofetil	16 (32.0%)	39.1%	25.9%
Rituximab	6 (12.0%)	8.7%	14.8%
**Maintenance therapy**			
Methotrexate	39 (78.0%)	78.3%	77.8%
Mycophenolate mofetil	32 (64.0%)	60.9%	66.7%
Rituximab	26 (52.0%)	43.5%	59.3%
Azathioprine	6 (12.0%)	8.7%	14.8%
**Newer therapies**			
Avacopan	9 (18.0%)	26.1%	11.1%
Tofacitinib	5 (10.0%)	17.4%	3.7%

**Note:** Percentages in the “*n* (%)” column are calculated with respect to the total cohort (*N* = 50). Percentages in gender columns are calculated with respect to the number of women (*n* = 23) or men (*n* = 27).

## Data Availability

The original contributions presented in this study are included in the article. Further enquiries can be directed to the corresponding author.
